# Cell cycle control and environmental response by second messengers in *Caulobacter crescentus*

**DOI:** 10.1186/s12859-020-03687-z

**Published:** 2020-09-30

**Authors:** Chunrui Xu, Bronson R. Weston, John J. Tyson, Yang Cao

**Affiliations:** 1 Genetics, Bioinformatics, and Computational Biology, Virginia Tech, Blacksburg, 24061 VA USA; 2 Department of Biological Sciences, Virginia Tech, Blacksburg, 24061 VA USA; 3 Department of Computer Science, Virginia Tech, Blacksburg, 24061 VA USA

**Keywords:** c-di-GMP, (p)ppGpp, *Caulobacter crescentus*, Nitrogen starvation, Cell cycle

## Abstract

**Background:**

Second messengers, c-di-GMP and (p)ppGpp, are vital regulatory molecules in bacteria, influencing cellular processes such as biofilm formation, transcription, virulence, quorum sensing, and proliferation. While c-di-GMP and (p)ppGpp are both synthesized from GTP molecules, they play antagonistic roles in regulating the cell cycle. In *C. crescentus*, c-di-GMP works as a major regulator of pole morphogenesis and cell development. It inhibits cell motility and promotes S-phase entry by inhibiting the activity of the master regulator, CtrA. Intracellular (p)ppGpp accumulates under starvation, which helps bacteria to survive under stressful conditions through regulating nucleotide levels and halting proliferation. (p)ppGpp responds to nitrogen levels through RelA-SpoT homolog enzymes, detecting glutamine concentration using a nitrogen phosphotransferase system (PTS ^Ntr^). This work relates the guanine nucleotide-based second messenger regulatory network with the bacterial PTS ^Ntr^ system and investigates how bacteria respond to nutrient availability.

**Results:**

We propose a mathematical model for the dynamics of c-di-GMP and (p)ppGpp in *C. crescentus* and analyze how the guanine nucleotide-based second messenger system responds to certain environmental changes communicated through the PTS ^Ntr^ system. Our mathematical model consists of seven ODEs describing the dynamics of nucleotides and PTS ^Ntr^ enzymes. Our simulations are consistent with experimental observations and suggest, among other predictions, that SpoT can effectively decrease c-di-GMP levels in response to nitrogen starvation just as well as it increases (p)ppGpp levels. Thus, the activity of SpoT (or its homologues in other bacterial species) can likely influence the cell cycle by influencing both c-di-GMP and (p)ppGpp.

**Conclusions:**

In this work, we integrate current knowledge and experimental observations from the literature to formulate a novel mathematical model. We analyze the model and demonstrate how the PTS ^Ntr^ system influences (p)ppGpp, c-di-GMP, GMP and GTP concentrations. While this model does not consider all aspects of PTS ^Ntr^ signaling, such as cross-talk with the carbon PTS system, here we present our first effort to develop a model of nutrient signaling in *C. crescentus*.

## Background

**Fig. 1:**
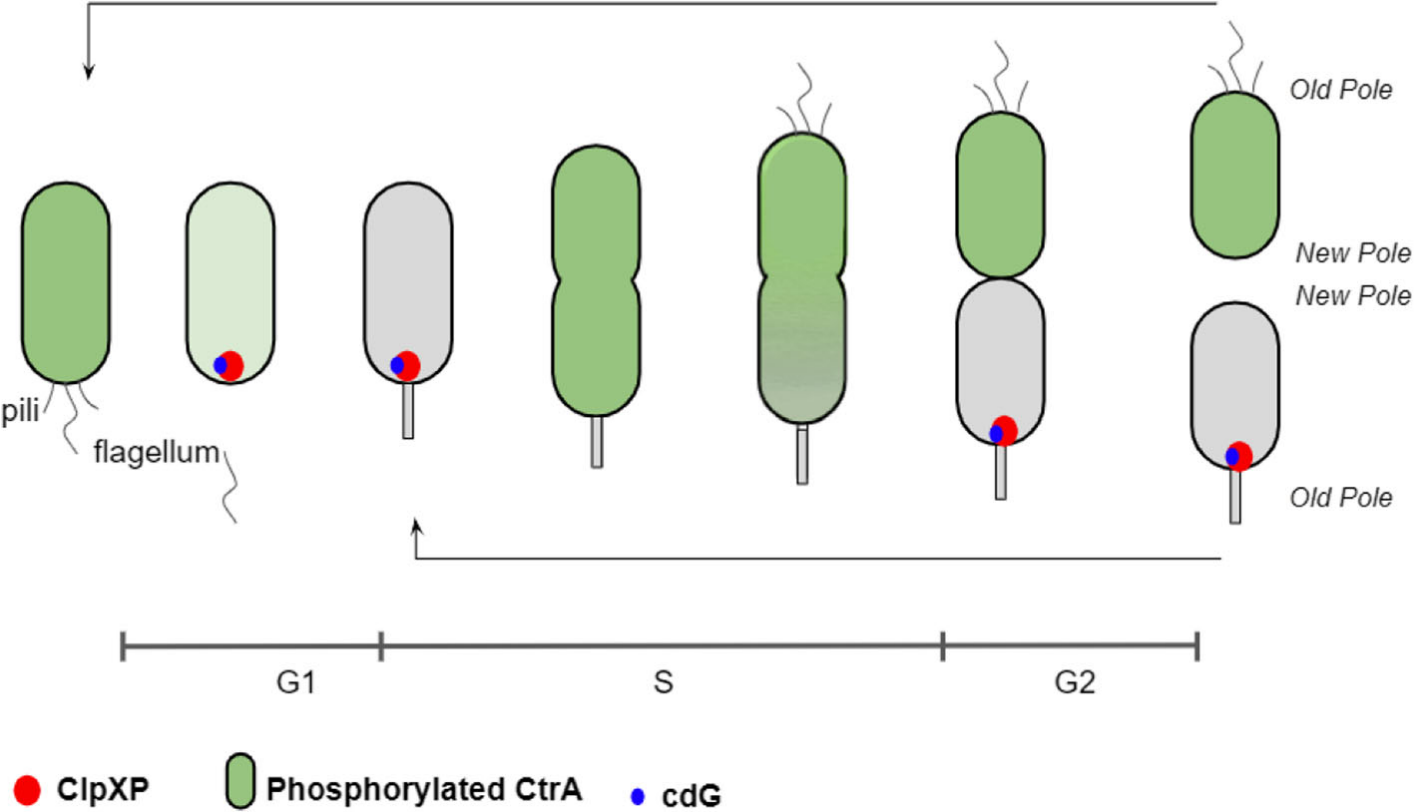
The asymmetric cell cycle of *C. crescentus*. A stalked cell is non-motile with a holdfast. A swarmer cell is motile with pili and a flagellum. A swarmer cell transforms into a stalked cell before DNA replication commences. CtrA regulates cell cycle progression in time and space. CtrA is eliminated during the G1-to-S transition. The green intensity indicates increasing concentrations of CtrA during cell cycle progression. ClpXP, a protease specific for CtrA, shows up at the old pole of a cell to degrade CtrA. c-di-GMP cooperates with ClpXP for CtrA proteolysis

*Caulobacter crescentus* is an oligotrophic, Gram-negative *α*-proteobacterium, frequently found in freshwater environments. *C. crescentus* undergoes asymmetric cell division, yielding two distinct progeny cells (Fig. 1): a non-motile ‘stalked’ cell (st) immediately re-enters the cell cycle and initiates DNA replication, while a motile ‘swarmer’ cell (sw) explores its environment before differentiating into a stalked cell and re-entering the cell cycle [1]. The stalked cell is equipped with a holdfast to attach to solid surfaces in its environment, whereas the swarmer cell develops a flagellum to move around in search of a suitable nutrient environment. The asymmetric cell cycle affords *C. crescentus* a certain flexibility to cope with the vagaries of life in an oligotrophic, aquatic environment [2].

Since asymmetric cell division plays an essential role in survival for *C. crescentus*, understanding how the asymmetry is regulated provides insight into the life cycle of many bacteria with similar characteristics. Many proteins, genes, and other molecules involved in the asymmetric pattern have been reported [2, 3]. CtrA, a master regulator of the *C. crescentus* life cycle, regulates more than 100 genes involved in flagellum biogenesis, DNA replication, and cell division [4, 5]. As CtrA inhibits the initiation of DNA replication, active CtrA (the phosphorylated form) must be eliminated during the swarmer-to-stalked (G1-to-S) transition. There are two pathways to inactivate CtrA: proteolysis by ClpXP [6] and dephosphorylation by CckA [7].

**Fig. 2:**
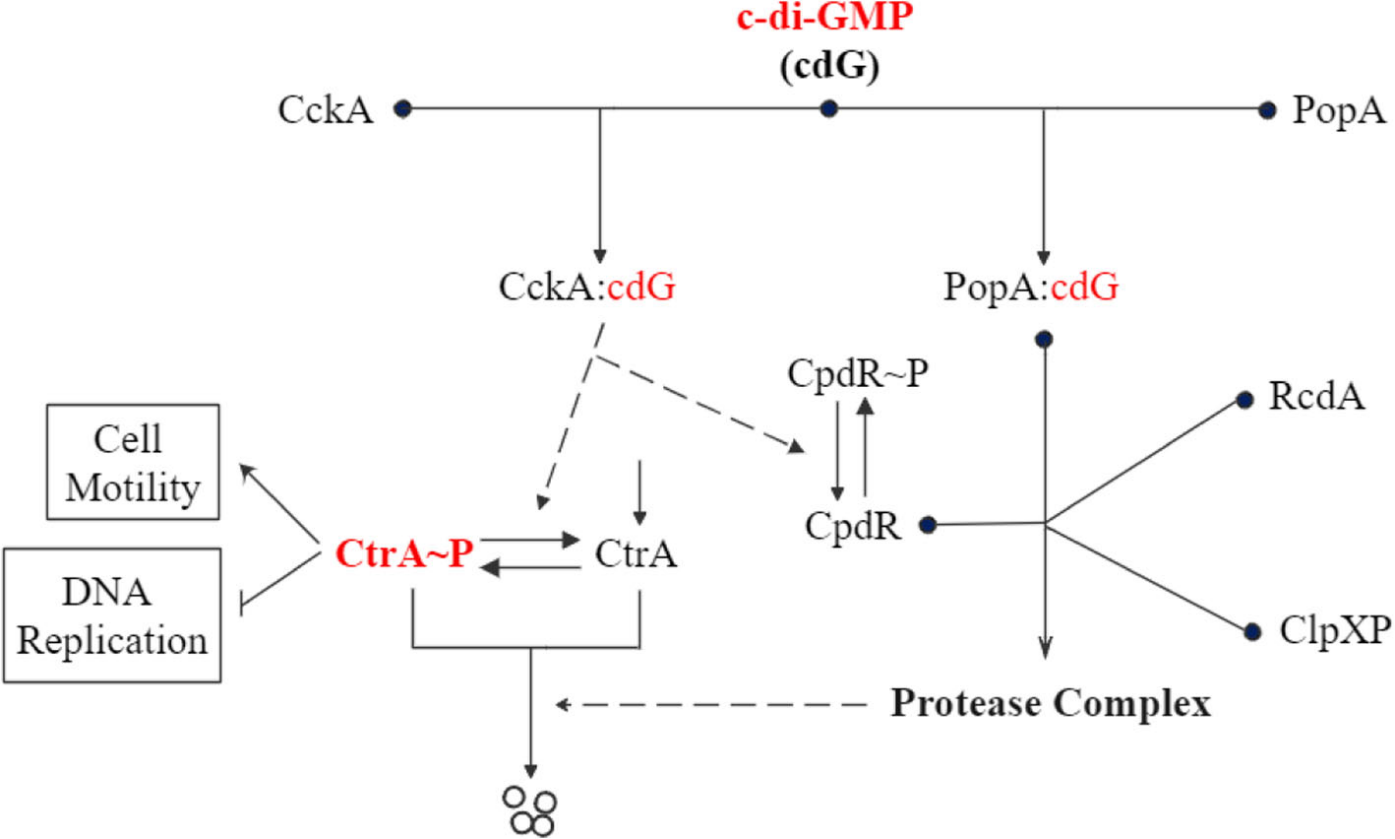
c-di-GMP regulates DNA replication and cell motility through CtrA. (Left-hand side) cdG directly stimulates phosphatase activity of CckA, thereby dephosphorylating CtrA to allow the initiation of DNA replication. (Right-hand side) cdG is also required for CtrA proteolysis

In *C. crescentus*, the spatio-temporally regulated proteolysis of CtrA requires protease ClpXP and additional factors called adaptors [6, 8]. The adaptor complex consists of CpdR, RcdA, PopA, and a second messenger c-di-GMP (cdG) (Fig. 2). ClpXP primed by unphosphorylated CpdR localizes at the old pole (Fig. 1) and recruits the adaptor RcdA which directly interacts with PopA. PopA must be bound with cdG to adapt CtrA to the entire protease complex (Fig. 2), which means cdG is indispensable for CtrA proteolysis. In addition to regulating CtrA proteolysis, cdG also participates in CtrA dephosporylation through CckA [7]. CckA is a bifunctional enzyme, which can act as both a phosphatase and a kinase to regulate CtrA and CpdR. When cdG binds with CckA, CckA activity favors the phosphatase state over the kinase state. When cdG level peaks during the G1-to-S transition, the dephosphorylation of CtrA and CpdR is rapidly stimulated, which allows DNA replication to initiate [9]. In this way, cdG stimulates DNA replication by activating the dephosphorylation and degradation of CtrA (Fig. 2).

While cdG stimulates the G1-to-S transition, alternative guanine-nucleotide based secondary messengers, guanosine tetraphosphate and guanosine pentaphosphate ((p)ppGpp), promote mobility and cell cycle arrest in *C. crescentus*. While the exact mechanisms are unknown, it is understood that (p)ppGpp indirectly promotes stablization of CtrA and degradation of DnaA, as well as interacting with RNA polymerase to influence global gene expression [10].

Additionally, cdG and (p)ppGpp control several key processes to help bacteria adjust to environmental cues, such as depletion of nutrients [10, 11]. There is evidence that bacteria accumulate (p)ppGpp in response to carbon and/or nitrogen limitation [10] by regulating RelA-SpoT homolog (RSH) enzymes. Furthermore, bacteria respond to the availability of carbon through cdG-regulated signaling processes [11]. However, the specific mechanisms by which stressful conditions affect cell cycle progression through the second messenger system and other key proteins in *C. crescentus* is not clear.

In this work, we combine cdG, (p)ppGpp, and GTP into one mathematical model to investigate the dynamics of these second messengers and how they respond to environmental changes through the PTS ^Ntr^. Our model suggests that the concentration of cdG decreases dramatically following nitrogen deprivation in response to increased synthetase and decreased hydrolase activity of the bifunctional enzyme, SpoT. This observation suggests a novel mechanism by which *C. crescentus* may regulate its cell cycle in response to nitrogen availability. Our model also suggests that (p)ppGpp-associated stability of CtrA may be a result of reduced cdG activity due to depletion of GTP. The dynamics of PTS ^Ntr^ enzymes have not yet been measured experimentally, however our model predicts how they might behave under various levels of nitrogen availability. Intracellular glutamine, phosphoenolpyruvate (PEP), and pyruvate (Pyr) affect the phosphorylation state of PTS ^Ntr^ enzymes in our model, which suggests that a stringent response to nutrient availability by guanine nucleotide-based second messengers may be enforced through both glutamine level and the concentrations of PEP and pyruvate.

## Methods

### Diagram construction

#### Metabolism and characterization of c-di-GMP

The cellular concentration of cdG is regulated by its synthesis by diguanylate cyclases (DGCs) and its degradation by phosphodiesterases (PDEs) [12]. DGCs (like PleD and DgcB), whose activities reside in the highly conserved GGDEF domain, act as dimers to produce cdG from two GTP molecules [13]. cdG negatively regulates its own synthesis by allosterically binding with the I-site of DGCs to inhibit synthetase activity [12].

**Fig. 3:**
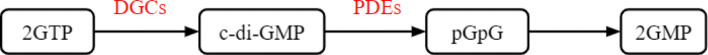
Schematic diagram of cdG metabolism. DGCs catalyze the synthesis of cdG. PDEs cleave cdG into pGpG, which is subsequently cleaved to two molecules of GMP

PDEs (such as PdeA and PdeB) cleave cdG to linear diguanylate (pGpG) or to GMP, based on the conserved EAL domain or HD-GYP domain, respectively [13]. As pGpG is eventually converted into GMP (Fig. 3), we ignore pGpG in the model and consider two molecules of GMP as the product of cdG degradation. In addition, the activity of some PDEs in *C. crescentus* is activated by binding GTP [14]. The initial velocity of hydrolysis by PDEs reaches V _max_/2 when the concentration of GTP is 4 *μ*M. Because GTP concentration in bacteria is much higher than 4*μ*M [15–17], we assume PDEs are constantly saturated with GTP and do not include this interaction in our model.

#### Metabolism and characterization of (p)ppGpp

**Fig. 4:**
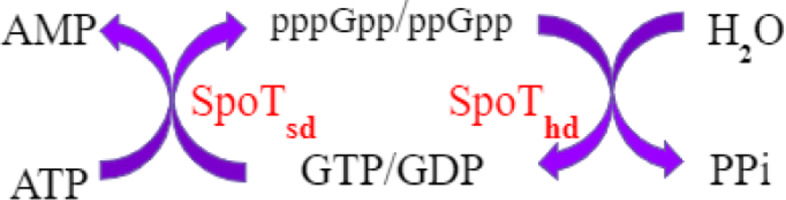
Metabolism of (p)ppGpp. SpoT, a bifunctional enzyme in *C. crescentus*, catalyzes both the synthesis and hydrolysis of (p)ppGpp

**Fig. 5:**
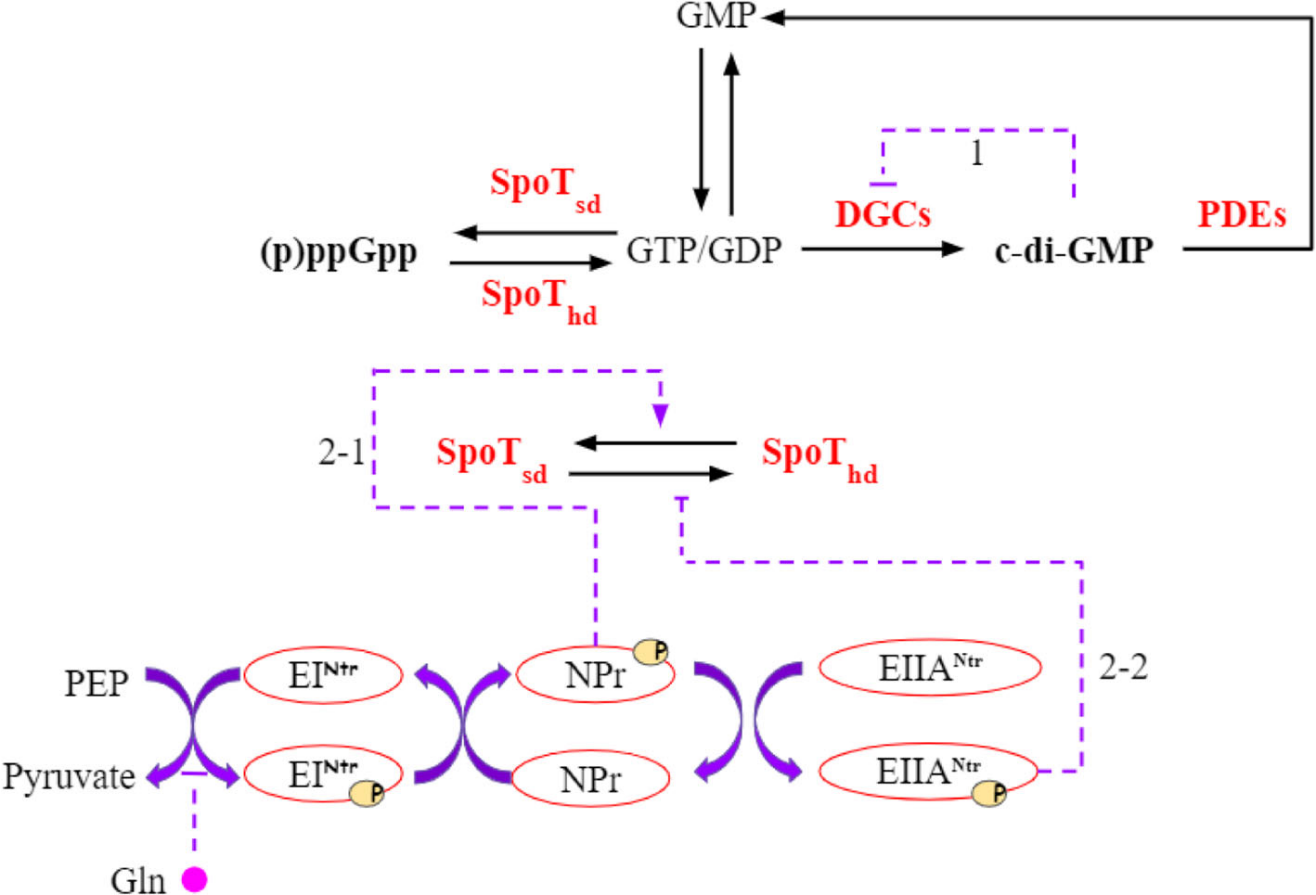
Diagram of our second messenger model. Glutamine (Gln) acts as the nitrogen signal, which regulates the phosphorylation state of PTS ^Ntr^ enzymes. Solid black arrows represent conversion between molecular species. Solid purple lines indicate transfer of phosphoryl groups between species. Phosphoryl transfer is reversible. Dashed lines represent allosteric influences on reaction rates (an arrow-head represents activation and a bar-head represents inhibition). Dashed line 1 indicates product-inhibition based on cdG binding to the I-site of DGCs. Dashed line 2-1 indicates that phosphorylated NPr indirectly activates the synthase activity of SpoT, and dashed line 2-2 indicates that phosphorylated EIIA ^Ntr^ directly inhibits the hydrolase activity of SpoT

(p)ppGpp accumulates in most bacteria under stressful conditions, such as nutrient starvation [10, 18]. In *C. crescentus*, (p)ppGpp delays the entry into S phase and the swarmer-to-stalked cell transition. This response gives *C. crescentus* an advantage in nutrient-deprived environments by maintaining its mobility to search for better environments and by delaying DNA replication to conserve energy [11]. *C. crescentus* utilizes the bifunctional enzyme SpoT, an RSH homologue, to catalyze the conversion between (GTP)GDP and (p)ppGpp [18–20] (Figs. 4, 5).

It has been reported that (p)ppGpp inhibits the synthesis of GMP and GDP through binding the corresponding synthetases, such as HPRT, GMK, and their homologues [21, 22]. The binding affinity of HPRT for pppGpp is *K*_d_ = 3.38 *μ*M in *E. coli*, but only 0.24 *μ*M in *C. crescentus* [22]. We ignored this inhibition (Fig. 5) because the HPRT homologue should be saturated with basal levels of (p)ppGpp in bacteria (10-50 *μ*M [11, 22]).

#### Nitrogen phosphotransferase system

It has been well documented that (p)ppGpp responds to carbon and nitrogen deprivations [11, 19, 21]. While the specific mechanism underlying carbon starvation is not yet clear, the mechanism responsible for nitrogen starvation has been recently elucidated [10]. The accumulation of (p)ppGpp following nitrogen starvation is regulated by the nitrogen phosphotransferase system (PTS^Ntr^) [10, 18].

The PTS ^Ntr^ consists of three components (EI^Ntr^,NPr,and EIIA^Ntr^) which form a phosphorylation cascade (Fig. 5). The first protein EI ^Ntr^ initiates the cascade through autophosphorylation using PEP as the phosphoryl donor. Then the phosphoryl group is transferred from EI ^Ntr^ to NPr and then to EIIA ^Ntr^. This process is reversible, so three components exchange phosphate groups and reach a steady state. EIIA ^Ntr^ can transfer its phosphate group to other unknown molecules [23]. We assume that the rate of phosphoryl transfer from EIIA ^Ntr^ to these other molecules outside of the PTS ^Ntr^ is far slower than the transfer rate among PTS ^Ntr^ proteins and the exchange with PEP and pyruvate. Therefore, we do not include a terminal phosphate sink in our model of the PTS ^Ntr^.

**Fig. 6:**
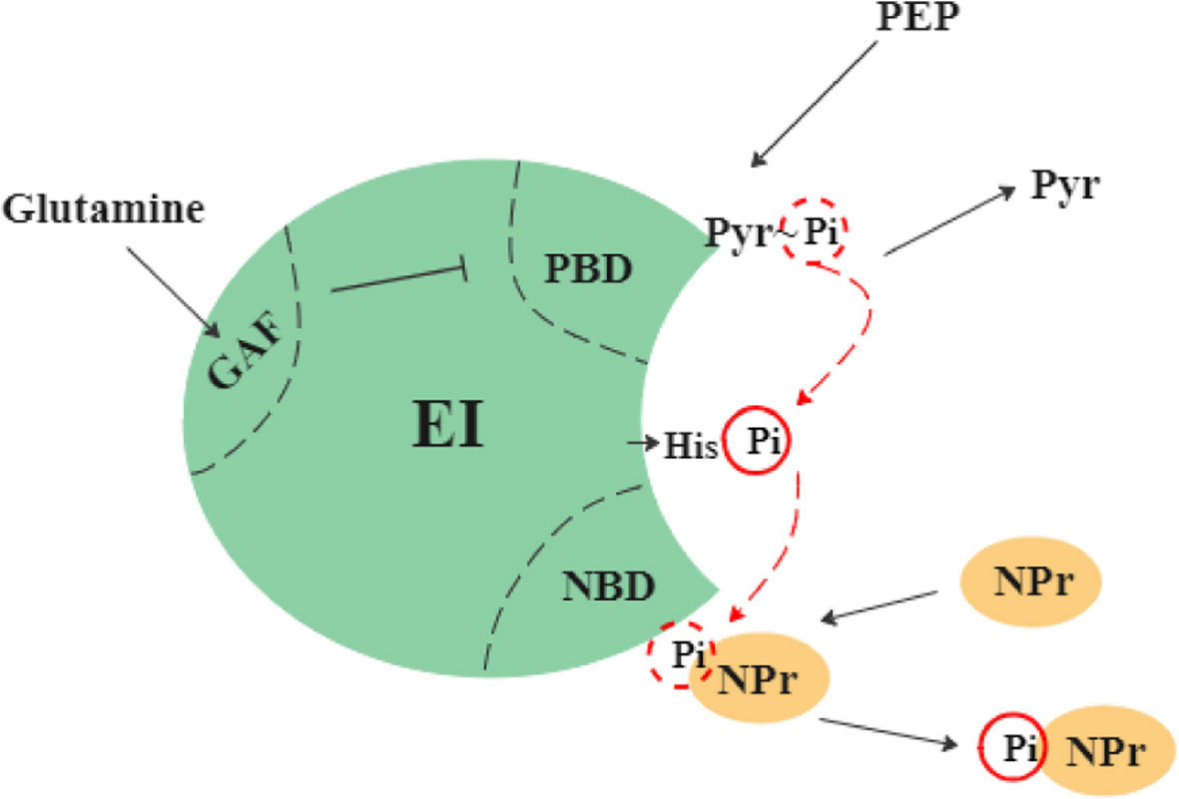
Schematic diagram of EI ^Ntr^ structure and phosphate transfer. The C-terminus of EI ^Ntr^ bears a PEP-binding domain (PBD) and the N-terminus is responsible for binding NPr (NBD). The red dashed arrows indicate the direction of phosphate transfer. The separate GAF domain senses nitrogen availability by binding glutamine, which inhibits phosphoryl group transferred from PEP to EI ^Ntr^

Glutamine binds to the conserved GAF domain of EI ^Ntr^ (Fig. 6) to prevent its autophosphorylation. Because glutamine works as a powerful nitrogen signal, enzymes involved in the PTS ^Ntr^ become highly phosphorylated under nitrogen starvation when the intracellular level of glutamine decreases rapidly [10]. The PTS ^Ntr^ influences cdG dynamics by its effects on SpoT activity. Bacterial two-hybrid assays and mutant experiments [18] indicate that phosphorylated EIIA ^Ntr^ directly interacts with SpoT to inhibit hydrolase activity, whereas phosphorylated NPr activates SpoT synthetase activity indirectly (Fig. 5). In this way, the PTS ^Ntr^, which senses nitrogen availability through glutamine, subsequently regulates SpoT activity and (p)ppGpp levels.

### Mathematical model

Based on the diagram in Fig. 5, the reactions of our model are as follows:

The activity of DGCs is subject to product inhibition through binding of cdG. As two cdG molecules bind allosterically to each DGC dimer, we assumed that cdG inhibition of DGC is a cooperative process. Thus we expressed the activity of [DGC] as a Hill function with a Hill exponent of 2 (Table 1, Eq. (1)). Unlike DGCs, PDEs act as monomers, which convert cdG to pGpG or GMP [13]. pGpG is subsequently converted into GMP [10]. We assumed this reaction is very fast and ignored the intermediate pGpG.

$$2\text{GTP} \stackrel{\text{DGCs}}{\longrightarrow} \text{c-di-GMP}\stackrel{\text{PDEs}}{\longrightarrow} 2 \text{GMP}. $$

**Table 1:** Equations of our mathematical model*

(1)	*d*[ cdG]/*dt*	=	$k_{\mathrm {s.cdG}}\cdot {\!\mathrm {[\!DGC]}}\cdot {\frac {K_{1}^{2}}{K_{1}^{2}+[\text {cdG}]^{2}}}\cdot {\frac {[\text {GTP}]^{2}}{[\text {GTP}]^{2}+K_{\mathrm {m1}}^{2}}}-k_{\mathrm {d.cdG}}\cdot {\mathrm {[\!PDE]}}\cdot {\frac {\mathrm {[cdG]}}{\mathrm {[cdG]}+K_{\mathrm {m2}}}}$
(2)	*d*[ (p)ppGpp]/*dt*	=	$k_{\mathrm {s.(p)ppGpp}}\cdot {\left \{\text {SpoT}_{\text {sd}}\right \}}\cdot {\frac {[\text {GTP}]}{[\text {GTP}]+K_{\mathrm {m3}}}}-k_{\mathrm {d.(p)ppGpp}}\cdot {\left \{\text {SpoT}_{\text {hd}}\right \}}\cdot {\frac {[\mathrm {(p)ppGpp}]}{[\mathrm {(p)ppGpp}]+K_{\mathrm {m4}}}}$
(3)	*d*[ GTP]/*dt*	=	$k_{\mathrm {s.GTP}}\cdot {[\text {GMP}]}-k_{\mathrm {d.GTP}}\cdot {[\text {GTP}]}-k_{\mathrm {s.(p)ppGpp}}\cdot {\left \{\text {SpoT}_{\text {sd}}\right \}}\cdot {\frac {[\text {GTP}]}{[\text {GTP}]+K_{\mathrm {m3}}}}$
			$+k_{\mathrm {d.(p)ppGpp}}\cdot {\left \{\text {SpoT}_{\text {hd}}\right \}}\cdot {\frac {[\mathrm {(p)ppGpp}]}{[\mathrm {(p)ppGpp}]+K_{\mathrm {m4}}}}-2\cdot {k_{\mathrm {s.cdG}}\cdot {\mathrm {[\!DGC]}}\cdot {\frac {K_{1}^{2}}{K_{1}^{2}+[\text {cdG}]^{2}}}\cdot {\frac {[\text {GTP}]^{2}}{[\text {GTP}]^{2}+K_{\mathrm {m1}}^{2}}}}$
(4)	*d*[ GMP]/*dt*	=	$2\cdot {k_{\mathrm {d.cdG}}\cdot {\mathrm {[PDE]}}\cdot {\frac {\mathrm {[cdG]}}{\mathrm {[cdG]}+K_{\mathrm {m2}}}}}+k_{\mathrm {d.GTP}}\cdot {[\!\text {GTP}]}-k_{\mathrm {s.GTP}}\cdot {[\!\text {GMP}]}$
(5)	*d*[ EI∼P]_tot_/*dt*	=	$k_{1}\cdot {\frac {K_{4}+\epsilon [\text {Gln}]}{K_{4}+[\text {Gln}]}}\cdot [\!\text {EI}^{\text {PEP}}]\,-\,k_{-1}\cdot \left [\!\text {EI}\!\sim \mathrm {P}^{\text {Pyr}}\right ]\!-k_{2}\cdot [\!\text {EI}\!\sim {\mathrm {P}}]_{\text {tot}}[\!\text {NPr}]+k_{-2}\cdot [\!\text {NPr}\!\sim {\mathrm {P}}][\text {EI}]]_{\text {tot}}$
(6)	*d*[ NPr∼P]/*dt*	=	*k*_2_·[ EI ∼ P]]_tot_[ NPr] −*k*_−2_·[NPr ∼ P][ EI]_tot_ − (*k*_3_·[ NPr ∼ P][ EIIA] −*k*_−3_·[ NPr][EIIA ∼ P])
(7)	*d*[ EIIA∼P]/*dt*	=	*k*_3_·[ NPr∼P][EIIA]−*k*_−3_·[ NPr][ EIIA∼P]
(8)	[EI][PEP]	=	$\phantom {\dot {i}\!}\!K_{{\mathrm {d}}_{1}}\cdot [\!\text {EI}^{\text {PEP}}]$
(9)	[ EI∼P][Pyr]	=	$\phantom {\dot {i}\!}\!K_{\mathrm {d}_{2}} \cdot \left [\!\text {EI}\sim {\mathrm {P}}^{\text {Pyr}}\right ]$
(10)	[ EI]_T_	=	[EI]+ [ E*I*^PEP^]+ [ EI∼*P*^Pyr^]+[EI ∼ P]
(11)	[ NPr]_T_	=	[ NPr]+[ NPr∼P]
(12)	[ EIIA]_T_	=	[ EIIA]+[ EIIA∼P]

^*^{SpoT$_{\text {sd}}\}= \frac {\alpha }{1+\alpha }$, {SpoT$_{\text {hd}}\}=\frac {1}{1+\alpha },\alpha =K_{\text {SpoT}}\cdot \frac {[\text {NPr} \sim \mathrm {P}]}{[\text {NPr} \sim \mathrm {P}]+K_{2}}/\frac {K_{3}}{[\text {EIIA} \sim \mathrm {P}]+K_{3}}$. {SpoT_sd_}and {SpoT_hd_}represent the fraction of total SpoT for synthetase and hydrolase, respectively

As GDP and GTP can be interconverted and their products, ppGpp and pppGpp, behave similarly [11, 19, 24], we lumped GDP and GTP into a single variable, ‘GTP’, and ppGpp and pppGpp are also condensed into one variable, (p)ppGpp. These ‘variables’ are interconverted by the synthetase and hydrolase activities of SpoT (SpoT _sd_ and SpoT _hd_, respectively). To take the direct and indirect effects of NPr ∼P and EIIA ^Ntr^ ∼P into consideration, we define a variable, *α* (Table 1), as the synthetase:hydrolase ratio of SpoT [25].



The interconversion of GTP, GDP and GMP is described compactly in our mathematical model by the reversible reaction



PEP binds to the C-terminal domain of EI and donates a phosphoryl group to His-189 (Fig. 6). Then the phosphoryl group is transferred to the next two enzymes, NPr and EIIA ^Ntr^, in sequence [26, 27]. Glutamine binds to an allosteric site of EI ^Ntr^ (the GAF domain) [28] and inhibits phosphoryl transfer to His-189 [10, 29]. The phosphorylation cascade is summarized by the following reactions:



where EI ∼P_tot_= EI ∼P + EI ∼P^Pyr^ and EI _tot_= EI + EI^PEP^. EI^PEP^ and EI ∼P^Pyr^ indicate EI bound with PEP and EI ∼P bound with Pyr, respectively. *k*_±*i*_ (*i*=1,2,3) are the rate constants of phosphorylation reactions, while $\phantom {\dot {i}\!}k_{\text {b}_{\pm j}}$ (*j*=1,2) are the rate constants of binding reactions.

Here, we make several assumptions to describe PTS ^Ntr^ reactions effectively:

1. As PEP binding to EI is a rapid process [26, 30], we assumed that PEP and Pyr binding reactions are much faster than phosphorylation reactions (reaching quasi-steady state) [26]. Therefore, we converted the binding reactions into algebraic equations (Table 1, Eqs. (8, 9)).
2. We assumed that EI ∼P and EI ∼P^Pyr^ phosphorylate NPr at the same rate, but EI ^Pyr^ is unstable and immediately dissociates into EI and Pyr. Similarly, we assumed that EI and EI ^PEP^can be phosphorylated by NPr ∼P, but EI ∼P^PEP^ is unstable and immediately dissociates into EI ∼P and PEP.
3. Since there is limited experimental data for the kinetic rate constants of the nitrogen PTS, we utilized experiments on the carbon PTS system to estimate these rate constants in our model. The kinetics of the carbon and nitrogen PTSs are likely very similar as they are homologues [31, 32].
4. The total concentrations of EI ^Ntr^, NPr and EIIA ^Ntr^ are assumed to be constants [33]. We estimated the total concentrations to be: [EI ^Ntr^] _T_ = 10 *μ*M, [NPr] _T_ = 30 *μ*M, and [EIIA ^Ntr^] _T_ = 30 *μ*M [34–36].

The resulting mathematical model consists of seven ODEs and five algebraic equations (Table 1 and Additional file 1). Parameters are defined in Table 2. Whenever possible, we estimated parameters from experimental data. Initial conditions in Table 3 were estimated from the intracellular concentrations in bacteria. The maximum concentration of cdG in *C. crescentus* is around 0.28 *μ*M [37]. The basal levels of (p)ppGpp and GTP in Gram-negative bacteria during normal conditions are around 50 *μ*M and 1000 *μ*M [15], respectively. The ratio of [(p)ppGpp] to [GTP] in *C. crescentus* varies from 0.15 to 1.9 under rich and limited nitrogen conditions [20, 38, 39]. We used these values to calibrate our model. The ODEs were solved in MATLAB with ode15s.

**Table 2:** Parameters

Parameter	Description	Source
*k*_s.cdG_=33.5/min	scaled synthesis rate of cdG	this study
*k*_d,cdG_=100/min	scaled degradation rate of cdG	this study
*K*_1_=0.5*μ*M	dissociation constant for product inhibition	[40]
*K*_m1_=1500 *μ*M	binding affinity of GTP	this study
*K*_m2_=0.06*μ*M	binding affinity of cdG	[40]
[DgcB]=0.7 *μ*M	scaled DgcB level	[41]
[basal PDEs]=0.2 *μ*M	scaled basal PDE level	this study
*k*_s.(p)ppGpp_=170*μ*M/min	synthesis rate of (p)ppGpp	this study
*k*_d.(p)ppGpp_=160*μ*M/min	degradation rate of (p)ppGpp	this study
*K*_2_=75*μ*M	binding affinity of NPr ∼P	this study
*K*_3_=10*μ*M	dissociation constant of EIIA ∼P	this study
*K*_SpoT_=4	constant of SpoT activity	this study
*K*_m3_=1000*μ*M	binding affinity of GTP	this study
*K*_m4_=2000*μ*M	binding affinity of (p)ppGpp	this study
*K*_4_=75.63*μ*M	parameters of glutamine inhibition	[42]
*ε*=0.1		
*k*_s.GTP_=1500/min	synthesis rate of GTP	this study
*k*_d.GTP_=100/min	degradation rate of GTP	this study
[ EI]_T_=10*μ*M	total enzymes levels	[34–36]
[ NPr]_T_=30*μ*M		
[ EIIA]_T_=30*μ*M		
*k*_1_=52.4/min	phosphotransfer constants	this study, [27, 42]
*k*_−1_=67.2/min		
*k*_±2_=1.2×10^4^/(min·*μ*M)		[27, 33]
*k*_±3_=3.7×10^3^/(min·*μ*M)		
$K_{\mathrm {d1}}=\frac {k_{b_{-1}}}{k_{b_{1}}}=350\mu \mathrm {M}$	dissociation constants	[27]
$K_{\mathrm {d2}}=\frac {k_{b_{-2}}}{k_{b_{2}}}=670\mu \mathrm {M}$

**Table 3:** Initial conditions

Variables	Initial Conditions (*μ*M)
c-di-GMP	0.3
GTP	1300
(p)ppGpp	100
GMP	20
EI ∼P	10
NPr ∼P	30
EIIA ∼P	30

## Simulations and results

### Oscillations of DGCs and PDEs

**Fig. 7:**
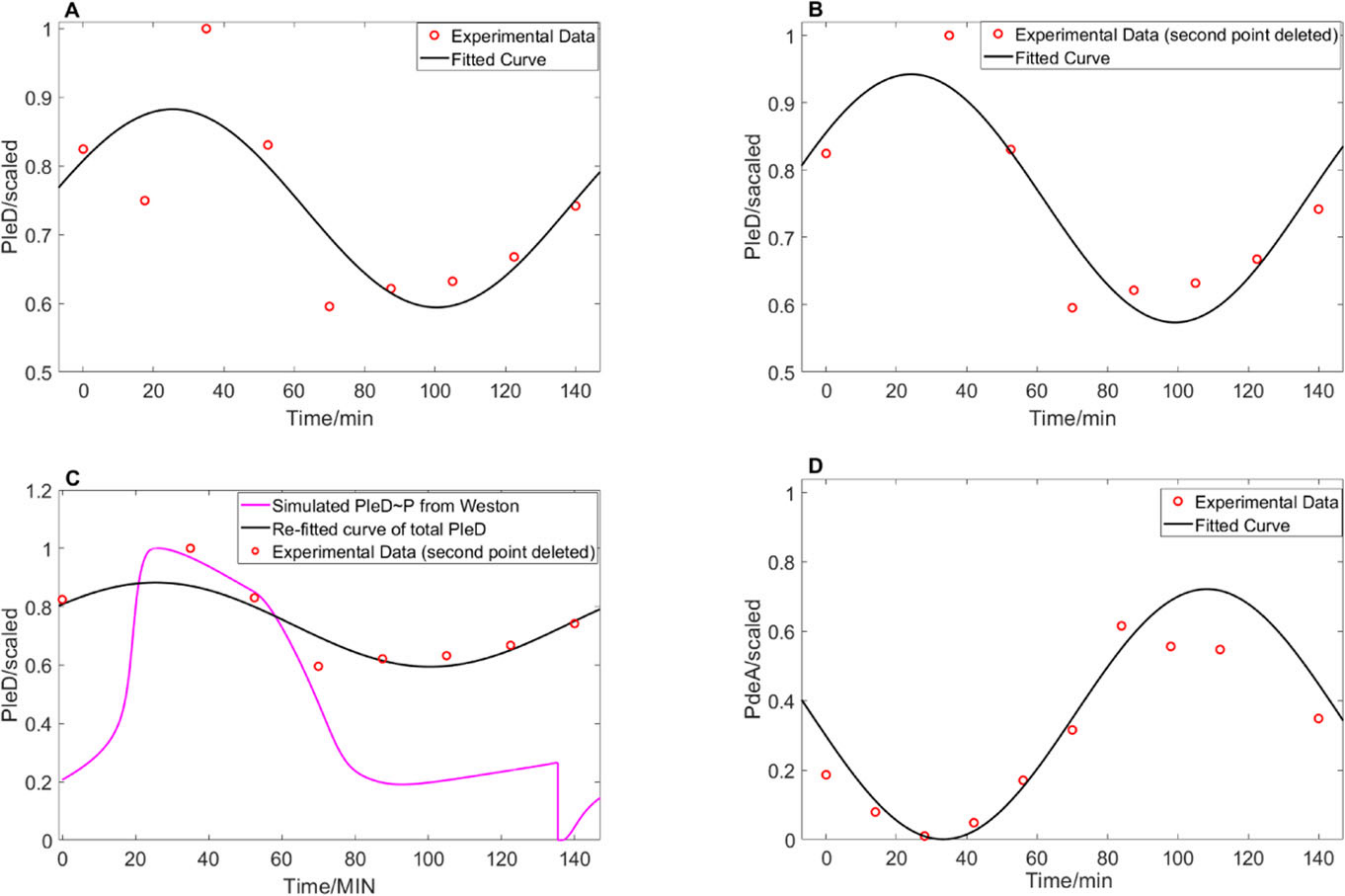
Curve-fitting of PleD and PdeA data. **a** Original experimental data of total PleD [41] and a curve fitted to the data by MATLAB. Function is 0.1442 ×sin$\left (\frac {\pi }{75}t+0.5037\right)$+0.7384. **b** Refitted PleD curve after deleting the second data point. New function is 0.1834 ×sin$\left (\frac {\pi }{75}t+0.5587\right)$+0.7579. **c** Weston’s simulation (unpublished) of phosphorylated PleD and comparison with re-fitted curve and experimental data from B. **d** Experimental measurements of PdeA [43] and its fitted curve. Function is -0.3605 × sin$\left (\frac {\pi }{75}t+0.1767\right)$+0.361

Two well-known DGCs in *C.crescentus* are DgcB and PleD [37, 41]. During progression through the cell cycle, DgcB level stays constant, but the concentration and activity of PleD vary [41]. Hence, we model [DGC] as the sum of constant [DgcB] and variable [PleD]. Because experimental data on the fluctuation of active (phosphorylated) PleD over the course of the *C. crescentus* cell cycle is not available, we used total PleD flucutations as a substitute. Figure 7a shows immunoblot measurements (red dots) of total PleD, extracted by ImageJ from [41], and the corresponding curve fitted by MATLAB (R-square is 0.66). It appears that the second data point from Abel et al. [41] is inaccurate because PleD activity should peak around t=20, since cdG needs to be produced at a high level at this time to deplete active CtrA and initiate the G1-to-S transition. Assuming that the second experimental point is an error, we re-fit the total PleD without this point (Fig. 7b, R-square is 0.84). In agreement with our expectations, the fitted curve in Fig. 7b increases during G1-to-S transition and peaks around 30 min. The corresponding accuracy of curve fitting improves as well. Additionally, we borrowed the active PleD simulation of an as-yet unpublished model by Bronson Weston (Fig. 7c, magenta curve) which captures the dynamics of phosphorylation of PleD. Weston’s simulation of PleD ∼P (Fig. 7c) shows a similar trend with the experimental data and re-fitted curve of total PleD (Fig. 7b,c), which serves to justify our methods for calibrating a curve for PleD activity. The different scaled levels between Weston’s simulation and experimental points are due to different normalization methods.

While PdeA is the most active phosphodiesterase enzyme in *C. crescentus* [44], other PDEs, including PdeB, PdeC, and PdeD, have been identified in bacterial species *B. subtilis*, *E. coli* and *L. monocytogenes* [45]. Assuming there are other PDEs in *C. crescentus* as well, we represented total [PDE] in our model as the sum of [basal PDE] plus a variable [PdeA] estimated by the curve-fitting tool in MATLAB applied to quantitative PdeA measurements derived from Western blots of [46] using ImageJ. The PdeA points and the corresponding curve are shown in Fig. 7d with R-square being 0.77.

### Oscillation of c-di-GMP over cell cycle in *C. crescentus*

**Fig. 8:**
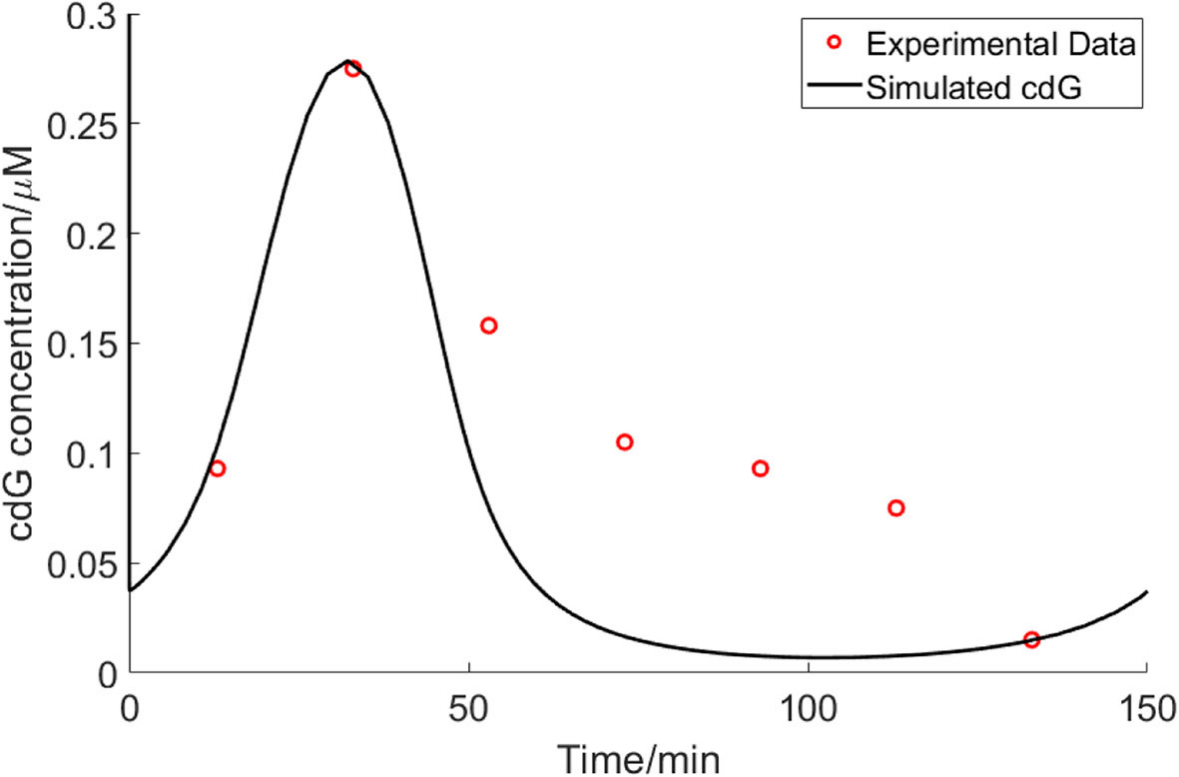
c-di-GMP oscillates during the cell cycle. Blue dots indicate experimental data of c-di-GMP in a single *C. crescentus* wild-type cell during one cell cycle [37]. Only c-di-GMP of a swarmer cell is shown. Black line indicates the simulated c-di-GMP in a swarmer cell at Gln=10000 *μ*M, PEP=300 *μ*M, and Pyr=1500 *μ*M

We used experimental data of cdG concentration (peak point) [37] and bacterial nucleotide concentrations to estimate parameters that are not available in publications. Experimentally, cdG peaks at the swarmer-to-stalked transition (≈0.28*μ*M) and then decreases until reaching the lowest value (<0.1*μ*M) in the swarmer cell after cell division. Our simulation of cdG over time fits experimental data well and shows a stable oscillation through the cell cycle under nutrient-rich conditions (Fig. 8), in agreement with experimental data [37].

### Comparison of simulated PTS ^Ntr^ to carbon-PTS experimental data

PTS and PTS ^Ntr^ have a lot in common. Enzymes of PTS ^Ntr^ (EI^Ntr^,NPr,and EIIA^Ntr^) are homologues of carbon-PTS enzymes (EI, HPr, and EIIA/B/C) [31]. They have similar structures and play parallel roles in nutrient uptake. In addition, these PTSs communicate with each other by phosphate exchange [28, 47]. PEP acts as the phosphoryl donor for both carbon and nitrogen PTSs. There are two significant differences between these systems [28]: (1) Enzymes II in PTS (juxtamembrane EIIB and transmembrane EIIC) assist in transmembrane transport of sugars [48] whereas PTS ^Ntr^ does not aid in sugar transport [32]. (2) PTS ^Ntr^ is regulated by glutamine levels as part of the nitrogen signaling pathway in prokaryotes, while PTS senses carbon sources in the environment through regulating transport and phosphorylation of carbohydrates like glucose [34, 49].

There is limited quantitative data for PTS ^Ntr^ in publications. Based on the similarities between PTS ^Ntr^ and PTS, we introduced some parameters obtained from PTS experiments to simulate PTS ^Ntr^ [27, 33] (Table 2). In order to calibrate the PTS ^Ntr^ model, we set [Gln] to 0 and compared simulations with carbon PTS experiments.

Kundig and Roseman [26, 50] measured how EI and HPr levels affect phosphorylation of PTS quantitatively (Table 4). We set our initial conditions to the experimental conditions of the paper, substituting [Npr] and [EI^Ntr^] for [Hpr] and [EI], respectively. As there is no information about pyruvate concentrations in their experiments, we estimated the [Pyr] based on one set of experimental data in Table 4. The Table indicates that our PTS ^Ntr^ simulations fit the experimental data well.

**Table 4:** Effect of EI and HPr (NPr) concentrations on phosphorylation of EI and HPr (NPr) in PTS system

Condition		Experiment^*^ [50]	Simulation
EI(*μ*M)	HPr(NPr)(*μ*M)	EI ∼P+HPr ∼P(*μ*M)	EI ∼P+NPr ∼P(*μ*M)
0.157	24.4	6	6.8
0.3125	24.4	6.5	6.9
0.729	24.4	7	7^**^
1.57	24.4	7.5	7.2
0.729	0	> 0 ^***^	0.2
0.729	12.2	3	3.6
0.729	36.6	9.1	10.5

^*^PEP=160 *μ*M

^**^This row has been used to estimate Pyr level; Pyr=48.5 *μ*M

^***^Too small to recognize the specific value from the original figure [50]

### Simulations under different nutrients conditions

Goodwin et al. [42] measured quantitatively how glutamine inhibits EI ^Ntr^ activity. In Table 5, we show model simulations under a range of glutamine levels. Lee et al. [51] showed that cellular glutamine in *E. coli* is very low under nitrogen-starvation and increases to more than 10000 *μ*M when environmental ammonium is increased. Therefore, in our simulations, we used [Gln]=1 *μ*M to represent limited nitrogen and [Gln]=10000 *μ*M to describe abundant nitrogen.

**Table 5:** Simulations under different conditions

	[Gln]	10000 *μ*M	2000 *μ*M	1000 *μ*M	100 *μ*M	10 *μ*M	1 *μ*M
	cdG range	0.01-0.28^*^	0.01-0.26	0.01-0.25	0-0.09	0-0.03	0-0.02^**^
	(p)ppGpp (*μ*M)	118	150	187	582	895	939
	GTP (*μ*M)	1221	1192	1156	787	493	452
[PEP]=300 *μ*M	$\frac {\text {[(p)ppGpp]}}{\text {[GTP]}}$	0.10	0.13	0.16	0.74	1.8	2.1
[Pyr]=1500 *μ*M	GMP (*μ*M)	81	79	77	52	33	30
	EI ∼ P (*μ*M)	0.5	0.6	0.8	2.0	3.2	3.4
	NPr ∼ P (*μ*M)	1.6	1.9	2.4	6.1	9.5	10.2
	EIIA ∼ P (*μ*M)	1.6	1.9	2.4	6.1	9.5	10.2
	cdG range	0.01-0.20	0.01-0.17^***^	0.01-0.13^***^	0-0.02	0-0.01	0-0.01
	(p)ppGpp (*μ*M)	294	371	**458**	993	1160	1178
	GTP (*μ*M)	1056	984	**902**	401	244	227
[PEP]=2800 *μ*M	$\frac {\text {[(p)ppGpp]}}{\text {[GTP]}}$	0.28	0.38	**0.51**	2.5	4.8	5.2
[Pyr]=900 *μ*M	GMP (*μ*M)	70	66	**60**	27	16	15
	EI ∼ P (*μ*M)	1.1	1.4	**1.6**	3.7	5.2	5.4
	NPr ∼ P (*μ*M)	3.4	4.2	**4.9**	11.1	15.6	16.3
	EIIA ∼ P (*μ*M)	3.4	4.2	**4.9**	11.1	15.6	16.3

^*^Proposed to be under condition of ammonia with high carbon

^**^Proposed to be under condition of nitrogen starvation

^***^Proposed to be under condition of ammonia with limited carbon

PEP, the phosphoryl donor of PTS ^Ntr^, is an important indicator of carbon availability [52]. However, Osanai et al. [53] and Yuan et al. [54] showed that PEP and Pyr levels are stable under nitrogen shifts. Hogema et al. [55] measured intracellular PEP and Pyr in *E. coli* under different carbon conditions: cells were initially grown in minimal medium (ammonia with limited carbon) and had PEP and pyruvate concentrations of 2800 *μ*M and [Pyr]=900 *μ*M, respectively. After adding 10mM glucose to the medium (now ammonia with high carbon), PEP and pyruvate concentrations shifted to 300 *μ*M and 1500 *μ*M, respectively. In consideration of this experimental data, we set ‘[PEP]=300 *μ*M, [Pyr]=1500 *μ*M, [Gln]=10000 *μ*M’ to represent ‘ammonia with high carbon’; and ‘[PEP]=300 *μ*M, [Pyr]=1500 *μ*M, [Gln]=1 *μ*M’ to represent ‘nitrogen-starved’ condition. As cells require carbon and nitrogen to synthesize glutamine, we regard limited glutamine (proposed as 1000-2000 *μ*M in Table 5) and ‘[PEP]=2800 *μ*M and [Pyr]=900 *μ*M’ as ‘ammonia with limited carbon’.

Table 5 summarizes the results of our simulations. cdG oscillations peak at 0.28 *μ*M under ’ammonia with high carbon’, and peak at 0.02 *μ*M under nitrogen depletion. These results suggest that depletion of nitrogen should result in cell cycle arrest, which is consistent with experimental observations [56]. In general, as glutamine concentrations decrease, cdG, GTP and GMP levels decrease while (p)ppGpp levels increase.

When we simulate conditions of ‘ammonia with limited carbon’, we find that cdG oscillations decrease in amplitude; however, concentrations are presumably not so low to induce cell cycle arrest. Thus, our results are consistent with the fact that *C. crescentus* continues to grow under such conditions [55]. Interestingly, our results suggest that (p)ppGpp levels should increase when decreasing carbon availability. Our results suggest that shifts in PEP and pyruvate concentrations due to limiting carbon availability will make the cell more sensitive to shifts in nitrogen. Table 5 suggests, that shifts in the direction of increased [PEP] and decreased [Pyr] favor increased activity in SpoT synthetase. Based on steady state analysis of ODEs in our model, phosphorylation of PTS ^Ntr^ proteins depends non-linearly on the [PEP]:[Pyr] ratio (Additional file 2). Increases in the ratio generally trend towards SpoT synthetase activity, while decreases trend toward hydrolase activity.

Our simulations show that the shift of c-di-GMP and (p)ppGpp levels in response to changes of nutrients is due to both a shift in internal glutamine concentration and an adjustment to the PEP and pyruvate levels (Table 5). Our model suggests that the PEP and Pyr levels regulating PTS ^Ntr^ is one potential pathway of (p)ppGpp response to carbon availability. Enzymes within the PTS ^Ntr^ system become highly phosphorylated under nitrogen starvation (Table 5), which is consistent with the existing qualitative analysis as well [10]. Additionally, our simulation fits the experimental observations (Table 6) well.

**Table 6:** Experimental information for concentrations and changes under starvation

Variables	Nutrient-rich	Nutrient-starved	Species	Reference
(p)ppGpp	50 *μ*M	-	*E.coli*	[11]
	10-30 *μ*M	millimolar	*B.subtilis*	[22]
GTP	900 *μ*M	-	*E.coli*	[16]
	-	3-fold drop (arginine starved)	*B.subtilis*	[21]
	1000-3000 *μ*M	-	*B.subtilis*	[17]
	-	3-fold drop (glucose starved)	marine *Vibrio*	[57]
GDP	100 *μ*M	-	*E.coli*	[16]
	-	14-fold drop (arginine starved)	*B.subtilis*	[21]
GMP	24 *μ*M	-	*E.coli*	[58]
[pppGpp]:[GTP] ratio	≈0.1	≈0.3 (arginine starved)	*B.subtilis*	[38]
	0.08	-	*E.coli*	[59]
[ppGpp]:[GTP] ratio	≈0.25	≈1.2 (arginine starved)	*B.subtilis*	[38]
	0.16	-	*E.coli*	[59]
	≈0.1	≈1.5 (arginine starved)	*C.crescentus*	[20]
		≈2.5 (glucose starved)		

### Response to environmental change

**Fig. 9:**
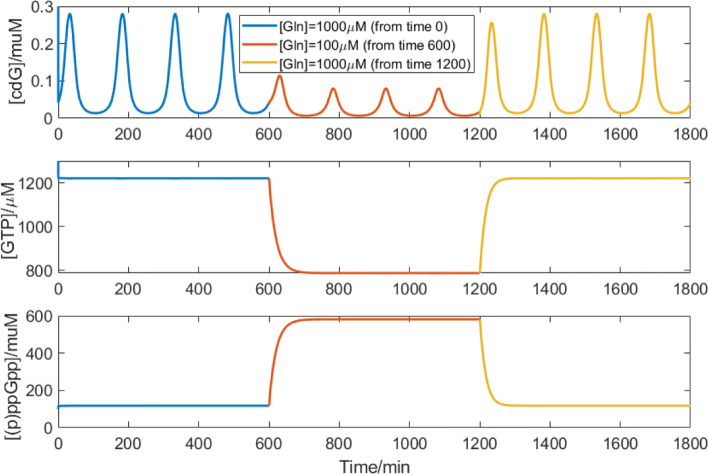
Response to nitrogen-shifts in our simulation. Blue line indicates simulated levels of cdG, GTP, and (p)ppGpp when [Gln]=10000 *μ*M. Red line indicates simulated levels of these three molecules when glutamine changes to 100 *μ*M at simulation time 600. Yellow line indicates the simulation when the glutamine recovers to 10000 *μ*M. The end point of previous simulation is used to be the initial point of the following simulation at a different glutamine level

Given the oligotrophic environments *C. crescentus* populates, we postulate that *C. crescentus* would have to respond rapidly to sudden shifts in nutrients in order to increase its fitness in these environments. Figure 9 shows how *C. crescentus* responds to environmental nitrogen-shifts in our simulation. The response time for starvation is within one cell replication cycle, which means *C. crescentus* can respond to nutrient deprivation quickly according to our model. Perhaps even more importantly, our model suggests that *C. crescentus* also recovers quite quickly to normal cell cycle oscillations when we reset glutamine to the starting value. A response within the time frame of one cell cycle would be a useful characteristic for *C. crescentus* to survive in oligotrophic environments.

## Discussion

Progression through the cell cycle in *C. crescentus* requires precise coordination of metabolic and morphological events. The guanine nucleotide-based second messenger network, including cdG and (p)ppGpp, plays significant roles in regulating bacterial morphology and metabolism, such as controlling the activity of CtrA and adapting bacteria to environmental changes. In this study, we propose a mathematical model to simulate the guanine nucleotide-based second messenger network, and investigate how this network responds to nutrient shifts through PTS ^Ntr^.

We calibrate our model using experimental data, and investigate two aspects influencing phosphorylation of PTS ^Ntr^ in simulations: 1) glutamine levels, which affects autophosphorylation of EI ^Ntr^ [10, 28]; and 2) PEP and Pyr levels, which influence the flux of phosphorus through the PTS ^Ntr^ system (Table 5, Additional file 2).

Simulations of nitrogen deprivation suggest that as (p)ppGpp accumulates in *C. crescentus*, GTP and cdG concentrations decrease significantly as a result of increasing SpoT synthetase activity. While it is suggested that (p)ppGpp stabilizes CtrA in *C. crescentus* [60], the exact mechanism is unknown. As cdG is essential for CtrA proteolysis, the stability of CtrA may increase due to diminished cdG concentration as a result of SpoT synthetase activity, rather than downstream effects of (p)ppGpp signaling. Thus, we propose that *C. crescentus* may respond to nitrogen starvation by stimulating the PTS ^Ntr^ system to induce SpoT synthetase activity, resulting in depletion of GTP and cdG levels to induce cell cycle arrest via stabilization of the chromosome replication inhibitor, CtrA.

Importantly, our results also suggest that changes to intracellular concentrations of PEP and pyruvate can have a significant impact on SpoT activity. We find that shifts in PEP and pyruvate concentrations in response to decreased sugar availability result in an increase in SpoT synthetase activity and an increase in sensitivity to shifts in glutamine concentration. Thus, two potential avenues to influence the PTS ^Ntr^ and SpoT are through adjusting PEP and pyruvate levels as well as glutamine.

## Conclusions and future work

Our mathematical model of guanine nucleotide-based second messenger network in *C. crescentus* (Fig. 5) agrees with experimental observations compiled from the literature. Most previous research has focused separately on cdG [14, 37] dynamics or the PTS ^Ntr^ system [10, 42] in bacteria, but has never related the second messenger network with the PTS ^Ntr^ system in order to study environmental impacts.

In this work, the interactions within the guanine nucleotide-based second messenger network are converted into a set of differential and algebraic equations (Table 1) to simulate second messenger response to nutrient conditions. cdG and (p)ppGpp, which play significant roles in cell cycle regulation, are connected with PTS ^Ntr^ to explain the relationship between bacterial development and environmental changes.

The current model consists of seven ODEs and five algebraic equations, describing the synthesis, degradation, activation, inhibition, phosphorylation, dephosphorylation, binding, and release of physiological variables in *C. crescentus*. Our resulting simulations are in good agreement with experiments and our model makes several intriguing predictions, however there are still several exciting directions that we can take this model:

1. To better understand how nitrogen signaling influences the cell cycle of bacteria, we can combine this second messenger model with a detailed regulatory model of the *C. crescentus* cell cycle [61]. It will be interesting to see if a shift in cdG concentration due to changes in SpoT activity will be enough to induce cell cycle arrest, or if intervention by (p)ppGpp is also necessary.
2. [PEP] and [Pyr] are adjustable signals in our model rather than variables. In the future, we hope to expand our model to include [PEP] and [Pyr] as dynamical variables to provide more insight into cell responses of nitrogen signaling.
3. Carbohydrate PTS catalyzes the uptake of carbohydrates [49], and it communicates with PTS ^Ntr^ through transfers of phosphoryl groups [62]. Therefore, including carbohydrate PTS into our model will help to understand environmental responses for both carbon and nitrogen shifts.

## Supplementary information

**Additional file 1** Calculations for ordinary differential equations.

**Additional file 2** Phosphorylation of PTS ^Ntr^ is non-linearly dependent on the [PEP]:[Pyr] ratio in simulation.

## Data Availability

The datasets generated and analysed during the current study are available at https://github.com/chunruixu/Second-messenger-model-in-Caulobacter.git

## Abbreviations

cdG: cyclic di-GMP, c-di-GMP
(p)ppGpp: alarmones guanosine tetraphosphate and guanosine pentaphosphate
PTS: phosphotransferase system
RSH: RelA-SpoT homolog
DGCs: diguanylate cyclases
PDEs: phosphodiesterases
Gln: glutamine
PEP: Phospho-enol-pyruvate
Pyr: Pyruvate
NDKs: nucleoside disphosphate kinases
