# Supplementary material for: Cell cycle control and environmental response by second messengers in Caulobacter crescentus
Source: BMC Bioinformatics. 2020 Sep 30;21(Suppl 14):408. doi: 10.1186/s12859-020-03687-z (PMC7526171; doi:10.1186/s12859-020-03687-z)
Supplement: Supplementary file 1 — Additional file 1 Calculations for ordinary differential equations. [file 12859_2020_3687_MOESM1_ESM.pdf]

Additional File 1: Calculations for Ordinary Differential Equations

A set of equations in Table 1 in the main text can be simplified further.

From Equation 8 and Equation 9, we can get the relationship between [EI] and $[\mathrm{EI}^{\mathrm{PEP}}]$, as well as $[\mathrm{EI} \sim \mathrm{P}]$ and $[\mathrm{EI} \sim \mathrm{P}^{\mathrm{Pyr}}]$:

$$\begin{array}{rcl} [\mathrm{EI}^{\mathrm{PEP}}] & = & \frac{[\mathrm{PEP}][\mathrm{EI}]}{K_{\mathrm{d1}}} \\ [\mathrm{EI}\mathrm{P}^{\mathrm{Pyr}}] & = & \frac{[\mathrm{Pyr}][\mathrm{EI}\sim\mathrm{P}]}{K_{\mathrm{d2}}} \end{array} \tag{A1.1}$$

We plug Equation A1.1 and Equation 10 into Equation 5 and write the expression as follows:

$$\begin{array}{rcl} \frac{d[\mathrm{EI} \sim \mathrm{P}]_{\mathrm{tot}}}{dt} & = & k_1 \cdot \frac{K_4+\epsilon[\mathrm{Gln}]}{K_4+[\mathrm{Gln}]} \cdot \frac{[\mathrm{PEP}][\mathrm{EI}]}{K_{\mathrm{d1}}} - k_{-1} \cdot \frac{[\mathrm{Pyr}][\mathrm{EI} \sim \mathrm{P}]}{K_{\mathrm{d2}}} \\ & - & k_2 \cdot [\mathrm{EI} \sim \mathrm{P}]_{\mathrm{tot}}[\mathrm{NPr}] + k_{-2} \cdot [\mathrm{NPr} \sim \mathrm{P}][\mathrm{EI}]_{\mathrm{tot}} \end{array} \tag{A1.2}$$

where $[\mathrm{EI}] = \frac{[\mathrm{EI}]_{\mathrm{T}} - [\mathrm{EI}\sim\mathrm{P}](1+\frac{[\mathrm{Pyr}]}{K_{\mathrm{d2}}})}{1+\frac{[\mathrm{PEP}]}{K_{\mathrm{d1}}}}$; $[\mathrm{EI}]_{\mathrm{tot}} = [\mathrm{EI}]_{\mathrm{T}} - [\mathrm{EI} \sim \mathrm{P}]_{\mathrm{tot}}$, $[\mathrm{EI} \sim \mathrm{P}]_{\mathrm{tot}} = [\mathrm{EI} \sim \mathrm{P}](1 + \frac{[\mathrm{Pyr}]}{K_{\mathrm{d2}}})$.

With the same method, we plug Equation 11 and Equation 12 into Equation 6 and Equation 7. Then we can rewrite ODEs A1.2, 6, 7 as follows:

$$\begin{array}{rcl} \frac{d[\mathrm{EI} \sim \mathrm{P}]_{\mathrm{tot}}}{dt} & = & k_1 \cdot \frac{K_4+\epsilon[\mathrm{Gln}]}{K_4+[\mathrm{Gln}]} \cdot \frac{[\mathrm{PEP}][\mathrm{EI}]}{K_{\mathrm{d1}}} - k_{-1} \cdot \frac{[\mathrm{Pyr}][\mathrm{EI} \sim \mathrm{P}]}{K_{\mathrm{d2}}} \\ & - & k_2 \cdot [\mathrm{EI} \sim \mathrm{P}]_{\mathrm{tot}}([\mathrm{NPr}]_{\mathrm{T}} - [\mathrm{NPr} \sim \mathrm{P}]) + k_{-2} \cdot [\mathrm{NPr} \sim \mathrm{P}][\mathrm{EI}]_{\mathrm{tot}} \\ \\ \frac{d[\mathrm{NPr} \sim \mathrm{P}]}{dt} & = & k_2 \cdot [\mathrm{EI} \sim \mathrm{P}]_{\mathrm{tot}}([\mathrm{NPr}]_{\mathrm{T}} - [\mathrm{NPr} \sim \mathrm{P}]) \\ & - & k_{-2} \cdot [\mathrm{NPr} \sim \mathrm{P}][\mathrm{EI}]_{\mathrm{tot}} \\ & - & k_3 \cdot [\mathrm{NPr} \sim \mathrm{P}]([\mathrm{EIIA}]_{\mathrm{T}} - [\mathrm{EIIA} \sim \mathrm{P}]) \\ & + & k_{-3} \cdot ([\mathrm{NPr}]_{\mathrm{T}} - [\mathrm{NPr} \sim \mathrm{P}])[\mathrm{EIIA} \sim \mathrm{P}] \\ \frac{d[\mathrm{EIIA} \sim \mathrm{P}]}{dt} & = & k_3 \cdot [\mathrm{NPr} \sim \mathrm{P}]([\mathrm{EIIA}]_{\mathrm{T}} - [\mathrm{EIIA} \sim \mathrm{P}]) \\ & - & k_{-3} \cdot ([\mathrm{NPr}]_{\mathrm{T}} - [\mathrm{NPr} \sim \mathrm{P}])[\mathrm{EIIA} \sim \mathrm{P}] \end{array} \tag{A1.3}$$

In this manner, the seven ODEs and five algebraic equations in Table 1 can be

rewritten rewritten as seven ODEs:

$$
\begin{aligned}
\frac{d[\mathrm{cdG}]}{dt} &= k_{\mathrm{s.cdG}} \cdot [\mathrm{DGC}] \cdot \tfrac{K_1^2}{K_1^2+[\mathrm{cdG}]^2} \cdot \tfrac{[\mathrm{GTP}]^2}{[\mathrm{GTP}]^2+K_{\mathrm{m1}}^2} \\
&- -k_{\mathrm{d.cdG}} \cdot [\mathrm{PDE}] \cdot \tfrac{[\mathrm{cdG}]}{[\mathrm{cdG}]+K_{\mathrm{m2}}} \\
\frac{d[\mathrm{(p)ppGpp}]}{dt} &= k_{\mathrm{s.(p)ppGpp}} \cdot \{\mathrm{SpoT_{sd}}\} \cdot \tfrac{[\mathrm{GTP}]}{[\mathrm{GTP}]+K_{\mathrm{m3}}} \\
&- k_{\mathrm{d.(p)ppGpp}} \cdot \{\mathrm{SpoT_{hd}}\} \cdot \tfrac{[\mathrm{(p)ppGpp}]}{[\mathrm{(p)ppGpp}]+K_{\mathrm{m4}}} \\
\frac{d[\mathrm{GTP}]}{dt} &= k_{\mathrm{s.GTP}} \cdot [\mathrm{GMP}] - k_{\mathrm{d.GTP}} \cdot [\mathrm{GTP}] - 2 \cdot k_{\mathrm{s.cdG}} \cdot [\mathrm{DGC}] \cdot \tfrac{K_1^2}{K_1^2+[\mathrm{cdG}]^2} \cdot \tfrac{[\mathrm{GTP}]^2}{[\mathrm{GTP}]^2+K_{\mathrm{m1}}^2} \\
&+ k_{\mathrm{d.(p)ppGpp}} \cdot \{\mathrm{SpoT_{hd}}\} \cdot \tfrac{[\mathrm{(p)ppGpp}]}{[\mathrm{(p)ppGpp}]+K_{\mathrm{m4}}} - k_{\mathrm{s.(p)ppGpp}} \cdot \{\mathrm{SpoT_{sd}}\} \cdot \tfrac{[\mathrm{GTP}]}{[\mathrm{GTP}]+K_{\mathrm{m3}}} \\
\frac{d[\mathrm{GMP}]}{dt} &= 2 \cdot k_{\mathrm{d.cdG}} \cdot [\mathrm{PDE}] \cdot \tfrac{[\mathrm{cdG}]}{[\mathrm{cdG}]+K_{\mathrm{m2}}} \\
&+ k_{\mathrm{d.GTP}} \cdot [\mathrm{GTP}] - k_{\mathrm{s.GTP}} \cdot [\mathrm{GMP}] \\
\frac{d[\mathrm{EI} \sim \mathrm{P}]_{\mathrm{tot}}}{dt} &= k_1 \cdot \tfrac{K_4+\epsilon[\mathrm{Gln}]}{K_4+[\mathrm{Gln}]} \cdot \tfrac{[\mathrm{PEP}][\mathrm{EI}]}{K_{\mathrm{d1}}} - k_{-1} \cdot \tfrac{[\mathrm{Pyr}][\mathrm{EI} \sim \mathrm{P}]}{K_{\mathrm{d2}}} \\
&- k_2 \cdot [\mathrm{EI} \sim \mathrm{P}]_{\mathrm{tot}}([\mathrm{NPr}]_{\mathrm{T}} - [\mathrm{NPr} \sim \mathrm{P}]) + k_{-2} \cdot [\mathrm{NPr} \sim \mathrm{P}][\mathrm{EI}]_{\mathrm{tot}} \\
\frac{d[\mathrm{NPr} \sim \mathrm{P}]}{dt} &= k_2 \cdot [\mathrm{EI} \sim \mathrm{P}]_{\mathrm{tot}}([\mathrm{NPr}]_{\mathrm{T}} - [\mathrm{NPr} \sim \mathrm{P}]) \\
&- k_{-2} \cdot [\mathrm{NPr} \sim \mathrm{P}][\mathrm{EI}]_{\mathrm{tot}} \\
&- k_3 \cdot [\mathrm{NPr} \sim \mathrm{P}]([\mathrm{EIIA}]_{\mathrm{T}} - [\mathrm{EIIA} \sim \mathrm{P}]) \\
&+ k_{-3} \cdot ([\mathrm{NPr}]_{\mathrm{T}} - [\mathrm{NPr} \sim \mathrm{P}])[\mathrm{EIIA} \sim \mathrm{P}] \\
\frac{d[\mathrm{EIIA} \sim \mathrm{P}]}{dt} &= k_3 \cdot [\mathrm{NPr} \sim \mathrm{P}]([\mathrm{EIIA}]_{\mathrm{T}} - [\mathrm{EIIA} \sim \mathrm{P}]) \\
&- k_{-3} \cdot ([\mathrm{NPr}]_{\mathrm{T}} - [\mathrm{NPr} \sim \mathrm{P}])[\mathrm{EIIA} \sim \mathrm{P}]
\end{aligned} \tag{A1.4}
$$
