# Supplementary material for: Cell cycle control and environmental response by second messengers in Caulobacter crescentus
Source: BMC Bioinformatics. 2020 Sep 30;21(Suppl 14):408. doi: 10.1186/s12859-020-03687-z (PMC7526171; doi:10.1186/s12859-020-03687-z)
Supplement: Supplementary file 2 — Additional file 2 Phosphorylation of PTS Ntr is non-linearly dependent on the [PEP]:[Pyr] ratio in simulation. [file 12859_2020_3687_MOESM2_ESM.pdf]

Additional File 2: Phosphorylation of PTS^{Ntr} is non-linearly dependent on the [PEP]:[Pyr] ratio in simulation.

We simulate $EIIA^{Ntr}$ ~P under various PEP and Pyr levels in Table A2.1 to investigate how [PEP], [Pyr], and [PEP]:[Pyr] ratio affect phosphorylation of PTS^{Ntr} proteins.

Table A2.1: Effect of [PEP]:[Pyr] ratio on phosphorylation of $EIIA^{Ntr}$ in simulation.

[$EIIA^{Ntr}$ ~P]μM / [PEP] \ [Pyr]	500μM	1000μM	1500μM	2000μM
500μM	3.1	2.3	2.0	1.8
1000μM	3.8	2.8	2.5	2.3
1500μM	4.1	3.0	2.7	2.5
2000μM	4.3	3.2	2.8	2.6

Based on steady state analysis of ODEs, $\frac{[\mathrm{EIIA}{\sim}\mathrm{P}]}{[\mathrm{EIIA}]}$, $\frac{[\mathrm{NPr}{\sim}\mathrm{P}]}{[\mathrm{NPr}]}$, and $\frac{[\mathrm{EI}{\sim}\mathrm{P}]_{\mathrm{tot}}}{[\mathrm{EI}]_{\mathrm{tot}}}$ are dependent on $\frac{[\mathrm{PEP}]^2(K_{\mathrm{d2}}+[\mathrm{Pyr}])}{[\mathrm{Pyr}]^2(K_{\mathrm{d1}}+[\mathrm{PEP}])}$ in our model, which means the phosphorylation fraction of PTS^{Ntr} proteins is non-linearly dependent with [PEP]:[Pyr] ratio.
